# Dynamic Neutrophil Subsets and Function in Lung Transplant Recipients: Insights from a One-Year Longitudinal Pilot Study

**DOI:** 10.3390/jcm14082660

**Published:** 2025-04-13

**Authors:** Naomi Kaisar-Iluz, Merav E. Shaul, Ofir Deri, Ella Huszti, Michael Peled, Yael Bezalel, Zvi G. Fridlender, Liran Levy

**Affiliations:** 1 Hadassah Medical Center, Institute of Pulmonary Medicine, Faculty of Medicine, Hebrew University of Jerusalem, Jerusalem 9112102, Israel; knaomi6@gmail.com (N.K.-I.); shaul.merav@gmail.com (M.E.S.); fridlender@hadassah.org.il (Z.G.F.); 2 Sheba Lung Transplant Program, Sheba Medical Center, Sackler Faculty of Medicine, Tel-Aviv University, Tel Aviv 6997801, Israel; ofir.deri@sheba.gov.il (O.D.); michael.peled@sheba.gov.il (M.P.); yael.bezalel@sheba.gov.il (Y.B.); 3 Biostatistics Research Unit, University Health Network, University of Toronto, Toronto, ON M5S 1A1, Canada; ella.huszti@thebru.ca

**Keywords:** lung transplant, neutrophil, plasticity, chronic lung allograft dysfunction

## Abstract

**Background**: Neutrophils are key innate immune cells in peripheral blood. In recent years, sub-populations of neutrophils have been identified. In addition to the normal-density neutrophils (NDNs) in both healthy subjects and patients, low-density neutrophils (LDNs) were described in chronic inflammation and cancer. In lung transplants (LTx), neutrophils play crucial roles in reperfusion injury, acute rejection, and chronic lung allograft dysfunction. Our pilot study examines neutrophil subsets and function in LTx recipients during the first post-transplant year. **Methods**: We collected blood from 11 LTx recipients at various intervals. LDNs and normal-density neutrophils (NDNs) were isolated. The production of reactive oxygen species (ROS) by NDNs was measured after PMA activation using a Luminol-HRP assay. Neutrophil phenotypic markers were analyzed with flow cytometry. **Results**: The LDN-to-NDN ratio increased at 3 and 6 months post-transplant. Expression levels of CD62-L (aging marker), PDL-1 (immune checkpoint), CD15 (maturation), and CXCR4 (homeostasis regulator) showed modulation. Interestingly, ROS production by NDNs was mildly elevated at baseline, reduced at 6 months, and returned to baseline levels by 9 months post-transplant. **Conclusions**: Neutrophils exhibit dynamic changes in the first post-LTx year. Investigating neutrophil plasticity could reveal clinically relevant biomarkers and facilitate the development of diagnostic and therapeutic tools in LTx.

## 1. Introduction

The intricate interplay between innate and adaptive immune responses plays a pivotal role in the realm of lung allograft immunity. The prevailing notion suggests that graft injury stemming from factors such as brain death, extended ischemia-reperfusion injury, infections, and other stimulants triggers an initial activation of the innate immune response. This, in turn, sets the stage for inflammation and primes the adaptive immune response. The latter comes into play at a subsequent phase, and notably, assumes dominance in donor antigen-specific alloresponses. Evidently, the innate immune system stands as a significant player in injury and governs the alloimmune reactions characteristic of organ transplantation [1,2].

While existing research has primarily spotlighted the deleterious actions of neutrophils in ischemia-reperfusion injury during lung transplant (LTx), where their oxidative and proteolytic effector activities fuel inflammation, their contributions extend beyond this domain [3,4]. Neutrophils wield a pivotal influence over adaptive immunity, as they possess the ability to migrate from peripheral sites to lymph nodes akin to antigen-presenting cells [5]. Through the expression of MHC and co-stimulatory molecules, they orchestrate T-cell differentiation and inflammation [6]. Neutrophils have emerged as significant participants in acute cellular rejection and antibody-mediated rejection, as well as chronic rejection and the complex process of inflammation resolution [7,8,9].

In non-transplant contexts, recent discoveries have shaken off the conventional belief that neutrophils are an undifferentiated cohort of general effector cells, serving merely as indicators of tissue inflammation. Instead, a paradigm shift has unfolded, revealing distinct neutrophil sub-populations with specialized roles under different physiological and pathological circumstances [10]. Surprisingly, the multifaceted nature of neutrophil heterogeneity and plasticity, especially within the context of organ transplantation, and in particular LTx, has yet to garner substantial attention within the transplant community. This pilot study aimed to characterize the behavior and attributes of circulating neutrophils, investigating potential subtypes and behavioral diversity among individuals who have undergone LTx during their first year following transplant.

## 2. Materials and Methods

### 2.1. Study Design and Patient Selection

This was a single-center cohort study approved by the Institutional Research Ethics Board. The study population was drawn from all consecutive, first, adult LTx recipients who underwent a bilateral LTx operation at Sheba Medical Center between 1 December 2020 and 30 December 2021. Patients with human leukocyte antibodies at the time of transplant were excluded. Patients who had two or fewer blood samples obtained were excluded as well. Follow-up data were obtained from electronic medical records and computerized databases and censored on 31 August 2023.

### 2.2. Clinical Management

#### 2.2.1. Surveillance Protocol

LTx recipients underwent scheduled surveillance bronchoscopies with bronchoalveolar lavage and transbronchial biopsies at 0.5, 1.5, 3, 6, 9, and 12 months post-transplant. Concurrent pulmonary function tests, donor-specific antibody testing, and chest computed tomography (starting at 3 months post-transplant) were performed.

#### 2.2.2. Immunosuppression Protocol

Maintenance immunosuppression: Induction immunosuppression was not routinely used. Standard immunotherapy consisted of tacrolimus (target levels: 10–15 ng/mL), mycophenolic acid at 1000–2000 mg/day, and methylprednisone at 0.5 mg/kg for three days, followed by prednisone at 0.5 mg/kg/d. This was gradually tapered to 0.25 mg/kg/d over three months and later generally tapered to 0.15 mg/kg/d at 6 months and 0.075 mg/kg/d at 12 months post-transplant.

Acute cellular rejection (ACR): Episodes of ACR were categorized utilizing the grading criteria outlined in the International Society for Heart and Lung Transplantation (ISHLT) guidelines [11]. Grade A1 episodes without clinical symptoms or significant changes in spirometry were not treated. Grade A1 episodes associated with clinical symptoms or grade A2 or higher were typically treated unless contraindicated by a concurrent infectious process. In addition to optimization of maintenance immunosuppression, the first-line treatment of ACR consisted of intravenous methylprednisolone at 10 mg/kg daily for 3 days, followed by a prednisone taper starting at 0.5 mg/kg/day and decreasing by 5 mg every five days back to the baseline dose. Patients with repeat ACR (i.e., accompanied by clinical symptoms, a drop in lung function, or grade A2 or higher) usually received repeat steroid boluses or a second-line treatment such as anti-thymocyte globulin (ATG). 

Chronic lung allograft dysfunction (CLAD): CLAD was defined as a sustained and irreversible decline in FEV1 to ≤80% of the post-transplant baseline, which was itself defined as the average of the two highest post-transplant FEV1 values measured at least three weeks apart, in the absence of other etiologies. When other etiologies were present, such as rejection or infection, but CLAD persisted after the resolution of these etiologies, the date of onset of CLAD was determined as the date of the first value of FEV1 ≤ 80% of the baseline in accordance with the 2019 consensus guidelines [12].

#### 2.2.3. Antimicrobial Prophylaxis

All patients received prophylaxis against Pneumocystis jirovecii Pneumonia with trimethoprim-sulfamethoxazole (800 + 160 mg thrice weekly) and 6 months of valganciclovir prophylaxis (900 mg daily, adjusted for renal function). Patients who were seronegative for CMV prior to transplant and who received an organ from a CMV-seropositive donor (D+/R−) were given 12 months of prophylaxis. Antifungal prophylaxis was not universally employed; instead, targeted antifungal therapy was initiated for patients deemed to be at greater risk for invasive fungal disease based on surveillance bronchoscopy results.

### 2.3. Human Blood Samples and Neutrophil Isolation

Blood samples were obtained at the time of the LTx operation and at 3, 6, 9, and 12 months post-transplant. Blood was overlaid on 3% dextran (Sigma-Aldrich, St. Louis, MO, USA) in 0.9% NaCl in a 1:1 ratio and left at room temperature (RT) for 35 min to allow erythrocyte sedimentation. The leukocyte-rich supernatant was loaded on top of Histopaque 1077 (Sigma-Aldrich, St. Louis, MO, USA) and centrifuged at 700× *g* for 30 min at RT, with no brake. Following centrifugation, normal-density neutrophils (NDNs) were collected in the pellet fraction (in the high-density fraction) with the red blood cells (RBCs) and a low-density fraction (LDF) collected from the 1077–plasma interface. RBC lysis was performed with water for 30 s and stopped by adding 5×PBS (Biological Industries, Beit HaEmek, Israel) supplemented with 2.5% BSA (ENCO, Tel Aviv, Israel).

#### 2.3.1. ROS Production

Purified NDNs were resuspended at a concentration of 1 × 10^3^/µL in Hank’s balanced salt solution without phenol red (HBSS) (Biological Industries, Beit Haemek, Israel) and placed in each well of a white 96-flat-bottom-well plate (Greiner bio-one, Kremsmünster, Austria). A luminol + horseradish peroxidase (HRP) (both from Sigma-Aldrich, St. Louis, MO, USA) stock solution in HBSS was added to each well to a final concentration of 50 µM luminol and 40 g/mL HRP. Cells were then treated with 20 nM PMA (Sigma-Aldrich, St. Louis, MO, USA), and chemiluminescence was determined for 1 h at 30 s intervals using a Tecan plate reader (InfiniteF200Pro, TECAN, Männedorf, Switzerland).

#### 2.3.2. Staining and Flow Cytometry

Following isolation, cells were resuspended in FACS buffer and then stained for 45 min on ice for various markers. The antibodies used include a-CD66b-Pacific Blue (Biolegend, San Diego, CA, USA), a-CD15-APC (Biogems, Westlake Village, CA, USA), a-CD10-APC (Biogems), a-CXCR4-APC (Biolegend), a-LOX-1-PE (Miltenyi Biotec, Gaithersburg, MD, USA), a-PD-L1-PE (Biolegend), and a-CD62L-PE (Biogems). First, the live cell population was gated, followed by gating of the CD66b+ population (neutrophils). Other markers were gated out from the CD66b+ population. PD-L1 and CD62L expression levels were further divided into high and low sub-populations. Immunostained cells were analyzed with a BD LSR-Fortessa cell analyzer (BD Biosciences, Franklin Lakes, NJ, USA) and data were processed with FlowJo X software (BD Biosciences, Franklin Lakes, NJ, USA).

### 2.4. Statistical Analysis

Demographic characteristics were summarized as counts and percentages for categorical variables and as standard measures (median and interquartile range (IQR)) for continuous variables. Overall comparisons between groups were conducted using one-way ANOVA tests, while pairwise comparisons using Tukey’s/Sidak’s tests accounted for multiple comparisons. Statistical significance was defined as a *p*-value less than or equal to 0.05. All analyses were conducted using GraphPad Prism software version 8 (GraphPad Software, San Diego, CA, USA).

## 3. Results

### 3.1. Study Cohort

The study cohort comprised eleven patients. Over a one-year post-transplant follow-up period, the investigation identified eleven instances of ACR (eight cases of A1 ACR, distributed across seven patients, and an additional three cases of A2 ACR in two different patients, of whom one also had A1 ACR). Notably, the cohort exhibited an absence of higher-grade ACR episodes and instances of AMR. One patient received a diagnosis of CLAD during the follow-up period. Comprehensive patient characteristics are delineated in Table 1 and Table 2.

### 3.2. LTx Increased the Ratio of LDNs to NDNs in Peripheral Blood

**Figure 1:**
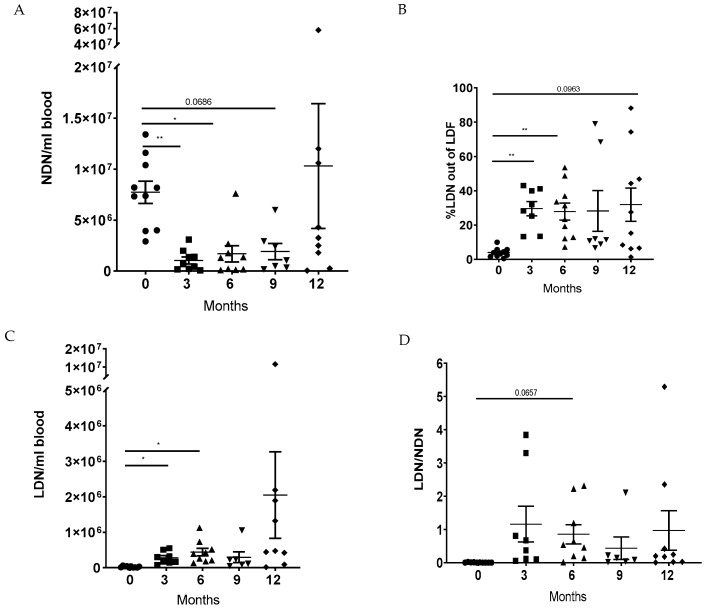
The proportion of circulating neutrophil sub-populations in lung transplant patients. NDNs and LDFs were isolated from the peripheral blood of patients at baseline (0), 3, 6, 9, and 12 months post-transplant. (**A**) NDNs were counted per 1 mL of blood. (**B**) The proportion of neutrophils in the LDF was quantified based on CD66b+ cells using flow cytometry. (**C**) Following the counting of total LDFs and based on the percentages of CD66B+ cells in the LDF, the amount of LDNs was calculated per 1 mL of blood. (**D**) The LDN/NDN ratio was calculated. Mean values ± SE are presented, n = 10. Statistical significance was determined by one-way ANOVA with the Tukey post hoc test. Statistical differences between each month are expressed with a star. * *p* < 0.05, ** *p* < 0.01.

The first aim of our study was to assess the proportion of neutrophil subsets, NDNs and LDNs, during the first year following LTx. We observed a decrease in the number of NDNs per 1 mL of blood during the 9 months post-transplant. Specifically, there was a significant reduction in NDN counts at 3 months (1.05 × 10^6^ ± 337.65 × 10^3^; *p* = 0.005) and 6 months (1.67 × 10^6^ ± 796.8 × 10^3^; *p* = 0.025) compared to the baseline count of 7.73 × 10^6^ ± 1.08 × 10^6^. NDNs were also reduced at 9 months, although this was not statistically significant. A tendency towards increased NDN counts was observed at one year post-transplant (Figure 1A).

In contrast, the percentage of LDNs in the low-density fraction (CD66b+) was significantly increased at 3 months (29.6% ± 4.2%; *p* = 0.002) and 6 months (27.9% ± 5%; *p* = 0.005) post-transplant, compared to baseline (4% ± 0.86%), with a tendency towards increased levels at 12 months (Figure 1B). Similar results were observed when measuring the amount of LDNs per 1 mL of blood. The amount of LDNs increased significantly at 3 months (0.28 × 10^6^ ± 61.36 × 10^3^; *p* = 0.019) and 6 months (0.44 × 10^6^ ± 106.17 × 10^3^; *p* = 0.023) compared to the baseline (0.25 × 10^6^ ± 6.7 × 10^3^) (Figure 1C).

Moreover, we found that the ratio of LDNs to NDNs increased over time, with a tendency towards an increase at 6 months (0.8571 ± 0.2908; *p* = 0.066) compared to the baseline (0.007 ± 0.002) (Figure 1D). These findings suggest that LTx has an impact on the number of neutrophils in the circulation, particularly by promoting the recruitment of LDNs and changing the LDN to HDN ratio.

### 3.3. Modulation of Neutrophils’ Phenotype Following LTx

**Figure 2:**
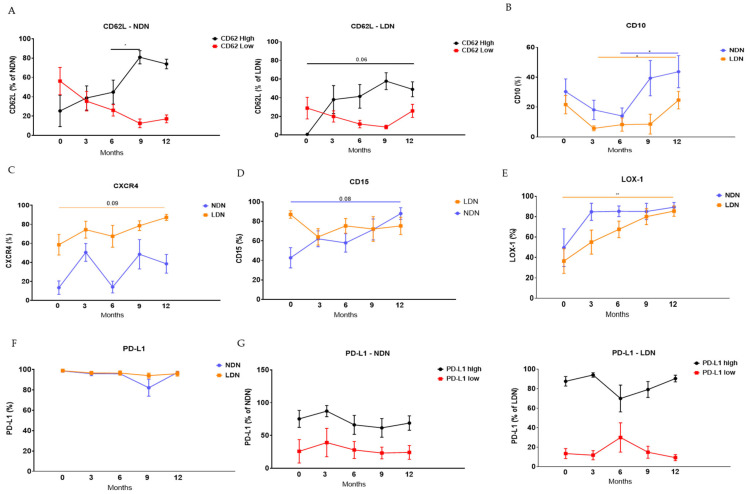
Phenotype of NDNs and LDNs following lung transplant. Following isolation of NDNs and LDFs from the circulation of the patients at baseline (0), 3, 6, 9, and 12 months post-transplant, the cells were stained for CD66b in combination with different neutrophil markers. (**A**) CD62L high vs. low in NDNs (left panel) and LDNs (right panel); (**B**) CD10; (**C**) CXCR4; (**D**) CD15; (**E**) LOX-1; (**F**) total PD-L1; (**G**) PD-L1 high vs. low in NDNs (left panel) and LDNs (right panel). The staining was assessed using flow cytometry. Mean values ± SE are presented, n = 11. Statistical significance was determined by one-way ANOVA with the Tukey post hoc test. Statistical differences between each month are expressed with a star. * *p* < 0.05, ** *p* < 0.01.

To investigate the effects of the LTx on the neutrophils’ phenotype, we conducted flow cytometry staining to evaluate the expression of several surface markers. Initially, we assessed the expression level of CD62L (a marker associated with neutrophil aging) by separating NDNs into two populations based on staining levels: CD62L-low (aged neutrophils) and CD62L-high (mature neutrophils). Our results demonstrated that over time, the relative expression of CD62L-high NDNs increased, while the expression of CD62L-low decreased, suggesting that the percentage of aged neutrophils in NDNs decreases. A significant increase in CD62L-high neutrophils was observed at 9 months (80.95% ± 6.86; *p* = 0.029) compared to 6 months (44.84% ± 12.41) post-transplant (Figure 2A, left panel). A similar trend was observed for the expression of CD62L-high by LDNs, with an increase from baseline (0.76% ± 0.76) to 12 months post-transplant (49.51% ± 8.77; *p* = 0.06). The expression of CD62L-low by LDNs did not show any significant difference over time (Figure 2A, right panel).

Next, we examined the expression levels of CD10, a marker associated with maturation, by NDNs and LDNs. The levels of CD10 mildly decreased until 6 and 9 months post-transplant but did not reach statistical significance. A significant increase in CD10 was observed at 12 months for both NDNs (43.68% ± 10.75; *p* = 0.032) and LDNs (24.67% ± 5.94; *p* = 0.035) compared to 6 months (14.07% ± 5.34) and 3 months (5.77% ± 1.69), respectively (Figure 2B), possibly suggesting a relative increase in mature neutrophils in the circulation.

Furthermore, we analyzed the expression levels of CXCR4, a chemotactic receptor, by NDNs and LDNs. This marker decreases on neutrophils during maturation. For NDNs, there was a tendency towards an increase at 3 months (50.48% ± 9.41) post-transplant compared to the baseline (13.37% ± 7.09), followed by a reduction at 6 months (14.07% ± 6.19) and an increase at 12 months (38.48% ± 9.83). The expression levels of CXCR4 by LDNs showed a tendency towards an increase at 12 months (87.21% ± 3.16; *p* = 0.09) compared to the baseline (58.47% ± 10.83) (Figure 2C). None of these changes reached statistical significance.

We also examined the expression levels of CD15, another maturation marker, by NDNs, which exhibited a mild non-statistically significant increase at 12 months (88% ± 6.03; *p* = 0.089) compared to the baseline (42.69% ± 10.38) (Figure 2D).

Finally, we assessed the expression levels of PD-L1, an immune checkpoint marker, and LOX-1, an immunosuppressive marker, by each sub-population of neutrophils. We divided the populations into PD-L1-low and PD-L1-high. However, there were no significant changes over time in the general positive population (Figure 2F) or in the levels of PD-L1 expression by NDNs (Figure 2G, left panel) or LDNs (Figure 2G, right panel). The expression levels of LOX-1 by NDNs tended to increase over time, although not significantly, while LDNs showed a significant increase of this marker at 12 months post-transplant (85.5% ± 5.11; *p* = 0.004) compared to baseline (36.52% ± 12.05) (Figure 2E).

### 3.4. LTx Affected ROS Production by NDNs

**Figure 3:**
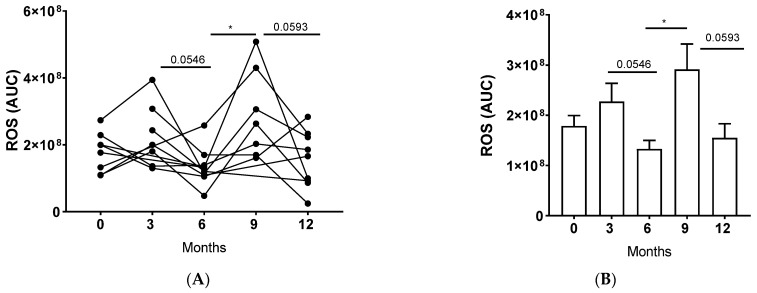
The impact of a lung transplant on neutrophils’ ROS production. NDNs were isolated from the peripheral blood of patients at baseline (0), 3, 6, 9, and 12 months post-transplant. ROS production was determined. Luminol, HRP, and 20 nM PMA were added to the neutrophils, and the appearance of chemiluminescence was measured over a time course of 1 h. The measured chemiluminescence corresponds to ROS production. AUC is presented. ROS production (AUC): (**A**) per patient and (**B**) per month. Mean values ± SE are presented, n = 10. Statistical significance was determined by one-way ANOVA with the Tukey post hoc test. Statistical differences between each month are expressed with a star. * *p* < 0.05.

We assessed a major function of neutrophils by examining ROS production. Remarkably, we observed modulation of ROS production by NDNs over time (Figure 3A,B). Initially, there was a tendency towards decreased ROS production at 6 months (1.33 × 10^8^ ± 17.03 × 10^6^; *p* = 0.055) compared to 3 months post-transplant (2.28 × 10^8^ ± 36.25 × 10^6^). Subsequently, there was a significant increase in ROS production at 9 months (2.92 × 10^8^ ± 50.45 × 10^6^; *p* = 0.034) compared to 6 months post-transplant. Finally, there was a tendency towards decreased ROS production at 12 months (1.55 × 10^8^ ± 28.14 × 10^6^; *p* = 0.059) compared to 9 months post-transplant (Figure 3B). Our findings suggest that LTx markedly affects the ability of NDNs to produce ROS over time. Unfortunately, the limited number of LDNs obtained from the available blood samples prevented us from assessing ROS production in this subset. Interestingly, previous studies in cancer patients have shown that while NDNs generate higher levels of ROS compared to LDNs, both populations exhibit similar responses to external stimuli [13,14].

## 4. Discussion

The intricate interplay and functional dynamics of neutrophils within the lifespan of the lung allograft have not yet undergone comprehensive investigation. To address this gap, we conducted a pilot study aiming to elucidate the behaviors and roles of neutrophils in LTx recipients throughout their first post-transplant year. Observations from the year-long monitoring of 11 recipients unveil significant alterations in neutrophil populations and characteristics. Notably, the ratio of LDNs to NDNs increases post-transplant, suggesting a chronic inflammatory situation, with LDN percentages and quantities rising significantly at 3 and 6 months following the LTx. Maturation and aging markers, including CD62L, CD10, and CD15, exhibit dynamic shifts, particularly evident at the 12-month interval. Notably, the expression patterns of LOX-1 and CXCR4 in neutrophils change over time, whereas PD-L1 expression remains consistently steady. There is variability in ROS production, indicating the influence of LTx and potential alterations in drug regimens on ROS generation. Collectively, our findings clearly indicate lung transplantation’s influence on neutrophil dynamics and phenotype, providing valuable insights for future research.

Neutrophils represent the predominant leukocyte population in the human circulatory system, constituting 50–70% of total circulating leukocytes [15]. Their vital role in host defense involves the phagocytosis and elimination of invading microorganisms through the release of cytokines, defensins, and reactive oxygen species [16]. For a considerable time, neutrophils were perceived as terminally differentiated effector cells; however, this notion has evolved over the past decade. Fridlender et al. demonstrated in murine models that tumor-associated neutrophils (TANs) within tumor tissue could be functionally divided into two distinct categories: N2 cells, which promote tumorigenesis by facilitating angiogenesis, tumor cell growth, and metastasis, and N1 cells that possess direct anti-tumor properties and the ability to directly eliminate tumor cells [17,18]. A crucial interplay between neutrophils and intratumoral CD8+ T cells was observed, alongside N2-TAN’s inhibition of lymphocyte activation and function [15]. A parallel diversity has been noted in circulating neutrophils in both murine cancer models and human cancer patients: NDNs exhibit anti-tumor traits, while LDNs adopt a tumor-permissive or even tumor-supportive role. Moreover, as tumors progress, a shift towards a tumor-permissive phenotype is observed, altering the balance between these neutrophil sub-populations [19]. These populations have been further described in several situations of chronic inflammation and infection, and the exact role of these changes is a developing and vibrant area of research [10].

Within the landscape of transplanted organs, compelling findings underscore the pivotal role played by neutrophils in orchestrating both acute and chronic inflammation regulation, often aligning their presence with tissue inflammation. However, emerging evidence has revealed the existence of neutrophil subsets, each uniquely equipped with specialized effector functions. These encompass the generation of neutrophils extracellular traps (NETs) and the facilitation of angiogenesis, thereby contributing to the intricacies of their roles [4].

The initial context within the realm of solid organ transplantation, where the involvement of neutrophils was first documented, pertains to acute allograft injury. One of the most damaging activities is the generation of ROS [20]. Neutrophil-mediated graft tissue damage is also driven by the release (degranulation) of tissue-digesting enzymes such as metalloproteinase-9 and neutrophil elastase [21]. Neutrophils can further stimulate graft inflammation by undergoing a distinctive programmed cell death process, giving rise to NETs, commonly referred to as “NETosis” [22]. Specifically, NETs have been reported in the BAL fluid of human LTx recipients with primary graft dysfunction [23], and a correlation has been described between the presence of NETs and primary graft dysfunction following LTx [24]. A clear connection has been noted between changes in ROS production and the level of NETs, suggesting a new target in neutrophil biology and disease [22,25].

Another dimension in which neutrophils exert their influence in LTx is through their participation in acute rejection. During acute cellular rejection, neutrophils establish direct interactions with different types of leukocytes, thus fostering alloimmunity within transplanted organs [26]. In the context of antibody-mediated rejection, clinical and pathological observations reveal a connection between graft neutrophilia and this type of rejection [27,28]. Unlike for T cell mediated-alloimmunity, there is comparatively little mechanistic data on how neutrophils regulate humoral responses to transplanted organs.

Shifting the focus to CLAD, the presence of infiltrating neutrophils is a frequently observed phenomenon in allografts undergoing chronic rejection. The prevailing perspective suggests that neutrophilia acts as an effector mechanism of IL-17 expression in chronic rejection, primarily driven by the accumulation of Th17 cells [29]. Furthermore, proteolytic enzymes commonly associated with extracellular matrix degradation and neutrophil migration, including metalloproteinases (such as MMP9 and MMP8), have been identified at elevated levels in patients affected by bronchiolitis obliterans syndrome (BOS), a form of CLAD [21]. Neutrophils might also contribute to persistent inflammation by influencing the maintenance of parenchymal tissues. A recent study demonstrated the buildup of neutrophil-derived alpha- and beta-defensin peptides in patients with BOS [30]. Intriguingly, a paradox arises, as maintaining a degree of neutrophilia may be imperative for the initiation or sustenance of tolerance within transplanted organs. Neutrophils can elicit anti-inflammatory signals in fellow phagocytes, concurrently releasing molecules that inhibit T-cell activation, further demonstrating their multifaceted functions [4].

The strength of this study lies in its introduction of a longitudinal perspective on patients throughout the first year after LTx, shedding light on the dynamics of neutrophils during this interval, independent of specific events such as infections or rejections. This unique vantage point offers an invaluable opportunity to elucidate the innate trajectory of neutrophils after LTx. Further enhancing its validity, this study benefits from a prospective design and was conducted within a proficient laboratory specializing in neutrophil research. While these findings provide insight into neutrophil dynamics after LTx, their direct clinical relevance remains to be determined. One of the key unanswered questions is whether these changes contribute to post-transplant rejection or CLAD development. Our study was not designed to establish causal relationships between neutrophil subsets and transplant outcomes. However, our findings raise the hypothesis that modifications in neutrophil function may play a role in the pathogenesis of CLAD, a possibility that warrants further investigation.

Despite these strengths, this study has several limitations. The small sample size restricts our ability to firmly link specific neutrophil behaviors to clinical outcomes. Additionally, subtle variations in patient care, such as adjustments to immunosuppressive regimens in response to ACR episodes or interventions for infections, could have influenced the results. However, overall patient management remained consistent. Another limitation is the absence of pre-transplant neutrophil profiling, including quantitative counts and defining characteristics, which could have served as a baseline reference.

In summary, this pilot study examined neutrophil behavior within the first year after LTx. The findings revealed significant changes in neutrophil populations, particularly increased ratios of low-density neutrophils and shifting maturation markers. These findings provide a foundation for future research on a larger scale, allowing for a more comprehensive understanding of neutrophil dynamics and their implications in the context of lung transplantation.

## Figures and Tables

**Table 1:** Baseline patient characteristics.

	N = 11
Baseline Characteristics	
Recipient age at transplant, year, median (IQR)	54 (47, 60)
Sex, male, n (%)	2 (18.2)
Native lung disease, n (%)	
Pulmonary fibrosis	6 (54.5)
Chronic obstructive pulmonary disease	1 (9.1)
COVID-19 ARDS	3 (27.3)
Bronchiectasis	1 (9.1)
CMV serology, n (%)	
D+R−	1(9.1)
D+R+	9 (81.8)
D−R+	1 (9.1)
D−R−	0
PGD at 72 h post-transplant, n (%)	
Grade 0	10 (90.9)
Grade 1	0
Grade 2	0
Grade 3	1 (9.1)
Acute rejection episodes during follow-up, n (%)	
AX	1 (1.5)
A0	48 (72.7)
A1	8 (12.1)
≥A2	3 (4.5)
Not done	6 (9.1)
Follow-up time, months, median (IQR)	27.6 (24.5, 30.9)
CLAD development during follow-up, n (%)	1 (9.1)
Death or retransplant during follow-up, n (%)	2 (18.2)

CLAD = chronic lung allograft dysfunction; CMV = cytomegalovirus; IQR = interquartile range; N = number; PGD = primary graft dysfunction.

**Table 2:** Acute cellular rejection and infection episodes during the first year post-transplant.

	2-Week Post-Tx		6-Week Post-Tx		3-Month Post-Tx		6-Month Post-Tx		9-Month Post-Tx		12-Month Post-Tx	
	AR Score	Infection	AR Score	Infection	AR Score	Infection	AR Score	Infection	AR Score	Infection	AR Score	Infection
1	A0BX	Y	A1BX	Y	A0BX	Y	A0BX	N	N	N	A0B0	Y
2	A2B0	Y	A0BX	Y	A0B0	Y	A0B0	N	A0BX	Y	A0BX	Y
3	A0BX	Y	A1BX	Y	A0BX	Y	A0BX	Y	A0B0	Y	A2B0	Y
4	A0BX	N	A0BX	N	A0BX	Y	A0B0	Y	A0B0	Y	A1B0	NA
5	A0BX	Y	A0BX	Y	A0BX	Y	NA	Y	NA	NA	NA	NA
6	NA	Y	A2BX	Y	A0B0	N	A1BX	Y	A0B0	Y	A2BX	Y
7	NA	Y	A0BX	Y	AXBX	Y	A0BX	Y	A0BX	Y	A0BX	Y
8	A0BX	Y	A0BX	Y	A0B0	Y	A0BX	Y	A0BX	Y	A1BX	Y
9	A1BX	N	A0BX	Y	A0BX	Y	A0BX	Y	A0BX	Y	A0BX	Y
10	A1BX	N	A0BX	N	A0B0	N	A0B0	N	A1BX	Y	A0BX	Y
11	A0BX	Y	A1BX	Y	A0BX	N	A0BX	N	A0B0	Y	A0BX	N

Abbreviations: AR = acute cellular rejection; NA = not available; N = no; Tx = transplant; Y = yes.

## Data Availability

The data that support the findings of this study are not openly available due to reasons of sensitivity and are available from the corresponding author upon reasonable request.

## Abbreviations

ACR	Acute Cellular Rejection
BOS	Bronchiolitis Obliterans Syndrome
CD	Cluster of Differentiation
CLAD	Chronic Lung Allograft Dysfunction
CMV	Cytomegalovirus
CXCR4	C-X-C Chemokine Receptor 4
FEV1	Forced Expiratory Volume in 1 s
HRP	Horseradish Peroxidase
IQR	Interquartile Range
LDN	Low-Density Neutrophil
LTx	Lung Transplant
MMP8	Matrix Metalloproteinase-8
MMP9	Matrix Metalloproteinase-9
NDN	Normal-Density Neutrophil
NETs	Neutrophil Extracellular Traps
PMA	Phorbol Myristate Acetate
ROS	Reactive Oxygen Species
